# The Interface of *Vibrio cholerae* and the Gut Microbiome

**DOI:** 10.1080/19490976.2021.1937015

**Published:** 2021-06-28

**Authors:** Jennifer Y. Cho, Rui Liu, John C. Macbeth, Ansel Hsiao

**Affiliations:** a Department of Microbiology and Plant Pathology, University of California, Riverside, CA, USA; b Department of Biochemistry, University of California, Riverside, California, USA; c Graduate Program in Genetics, Genomics, and Bioinformatics, University of California, Riverside, California, USA; d Division of Biomedical Sciences, School of Medicine, University of California, Riverside, California, USA

**Keywords:** *Vibrio cholerae*, virulence factors, microbiome, quorum sensing, bile salts, T6SS, nutrition, oral cholera vaccines

## Abstract

The bacterium *Vibrio cholerae* is the etiologic agent of the severe human diarrheal disease cholera. The gut microbiome, or the native community of microorganisms found in the human gastrointestinal tract, is increasingly being recognized as a factor in driving susceptibility to infection, *in vivo* fitness, and host interactions of this pathogen. Here, we review a subset of the emerging studies in how gut microbiome structure and microbial function are able to drive *V. cholerae* virulence gene regulation, metabolism, and modulate host immune responses to cholera infection and vaccination. Improved mechanistic understanding of commensal–pathogen interactions offers new perspectives in the design of prophylactic and therapeutic approaches for cholera control.

## Introduction

*Vibrio cholerae* is a Gram-negative bacterium and the etiologic agent of the severe human diarrheal disease cholera. Cholera affects millions of individuals yearly, and causes over 100,000 deaths per year.^1^ The voluminous watery diarrhea and vomiting characteristic of cholera can rapidly lead to severe dehydration, hypovolemic shock, and death if left untreated, with case-fatality rates in excess of 50%.^2^ While the development of oral rehydration therapy has dramatically reduced the treated case fatality, cholera continues to represent a severe global health and economic challenge,^3^ and thus demands better prophylactic and therapeutic interventions.

In between epidemics in human populations, *V. cholerae* persists in aquatic environments such as rivers, estuaries, and coastal waters, often in association with zooplankton, copepods, and other marine organisms. Toxigenic *V. cholerae* can then spread from these environments into human populations through contamination of water and food sources. In the human host, *V. cholerae* preferentially colonizes the epithelium of the distal small intestine. Once there, this pathogen reacts to a number of environmental cues to produce cholera toxin (CT), and the toxin-coregulated pilus (TCP). TCP is critical for colonization of the gut epithelium, while CT alters host cell signaling pathways leading to cellular damage and the profuse watery diarrhea characteristic of cholera, which aids in the dissemination of the pathogen back into the environment to continue the infection cycle.

In the host intestine, *V. cholerae* must respond to environmental signals in order to regulate the virulence-associated genes to drive colonization, survival, and host interaction. Emerging research suggests a key role for the commensal microbial community of the gastrointestinal tract, the gut microbiome, in these interactions. The gut microbiome is thought to outnumber human somatic cells, and encodes a bewildering array of biochemical functions that shape the gut environmental milieu for pathogenic and commensal microorganisms alike.^4,5^ All domains of life are represented in the diverse community of the gut microbiome, though the gut microbiome is dominated by the eubacteria. Of all the body sites to host commensal microorganisms, the gastrointestinal tract is by far the most densely colonized. This gut microbial community varies dramatically from host species to host species, from individual human to individual human, and can rapidly re-configure in response to environmental changes such as dietary change, malnutrition, diarrhea, or antibiotics.^6–11^ Early work by Freter et al. in the 1950s showed a role for interactions with gut commensal microorganisms in driving *V. cholerae* behavior in the gut; rodents whose commensal microflora had been depleted with antibiotics were far more susceptible to *V. cholerae* colonization, in contrast to untreated animals that remained strongly resistant.^12^ In contrast, *V. cholerae* is able to colonize the proximal and distal intestines of germ-free mice to high density.^13^

**Figure 1.**
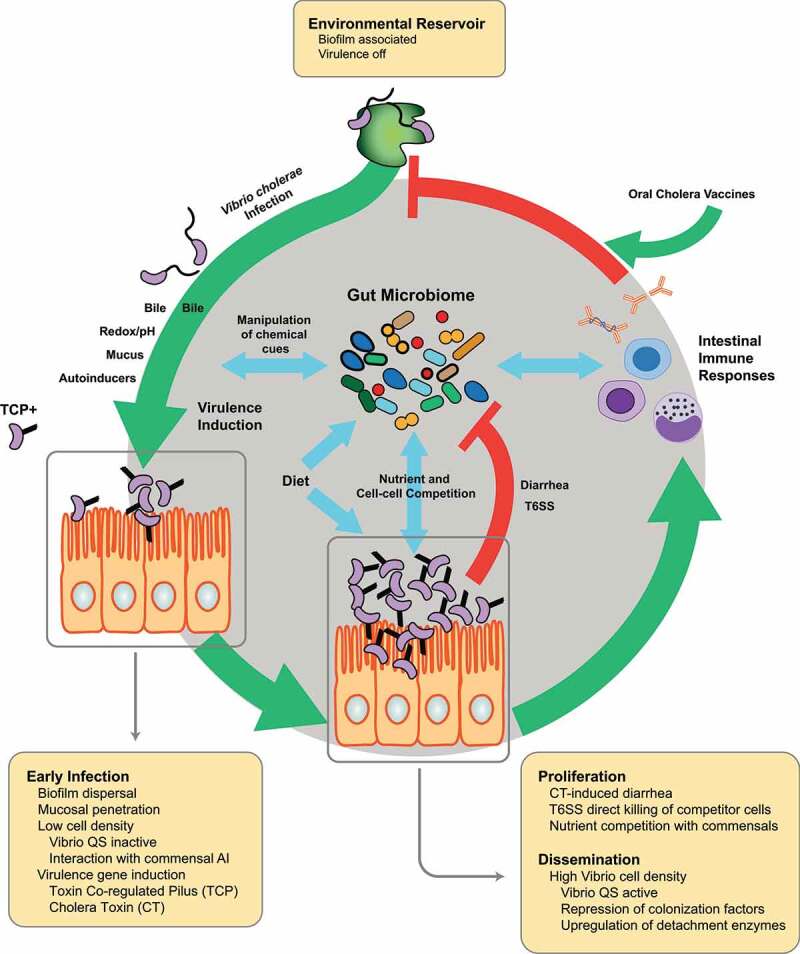
**The life and infection cycle of *V. cholerae* and its interaction with the gut microbiome**. The gut microbiome interacts with multiple aspects of *V. cholerae* pathogenesis. *V. cholerae* persists in environmental reservoirs, often in the form of biofilms that promote environmental persistence and protection from host gastric acid barriers on infection. The transition into human communities leads to coordinated changes in gene expression modulated by environmental factors such as bile, redox, pH, mucus, and quorum sensing, which are all influenced by the metabolism of gut commensals. This process regulates the expression of key virulence factors such as cholera toxin (CT) and the toxin coregulated pilus (TCP) that are required for diarrhea and colonization respectively. In early infection, *V. cholerae* disperse from biofilms. At low *V. cholerae* cell density in this phase, quorum sensing is inactive and virulence gene expression is active, subject to interaction with commensal-derived autoinducer molecules that can subvert this regulatory process. As *V. cholerae* proliferates, environmental changes in the gut promoted by diarrhea and other factors, alongside type VI secretion activity (T6SS) and nutrient competition, contributes to the clearance of competitor microbes. During late infection, the density of *V. cholerae* cells and quorum sensing molecules is high, leading to repression of virulence and upregulation of factors associated with detachment from the intestinal mucosa and dissemination into the environment. Host immunity, whether from previous natural infection or oral cholera vaccination, can modulate the susceptibility of subsequent hosts to infection from disseminated *V. cholerae*. The diversity of metabolic functions encoded by the gut microbiome determines several aspects of this cycle, including the timing of coordinated gene expression in different phases of infection, competition and persistence *in vivo*, and immune response. The interpersonal variation of the microbiome and the biochemical functions of the commensal microbial community of the gut may thus serve as potent determinant of *V. cholerae* behavior in human populations

Recent advances in gnotobiotic animal models with defined microbial content and multi-omics approaches in both human and animal studies have dramatically expanded our ability to examine the diversity and function of these microbes in the intestine, and to mechanistically dissect drivers of microbiome diversity and how microbiome diversity in turn interacts with invading microbes such as *V. cholerae*. This work suggests that differences in the microbiome, which is highly diverse from individual to individual, may drive personalized outcomes to *V. cholerae* infection through multiple mechanisms, and define several emerging areas of commensal-pathogen-host interaction research, including (i) microbiota-mediated manipulation of chemical signaling in the gut, (ii) the role of diet and nutrition in inter-microbial interactions during infection, and (iii) modulation of host immunity after infection and vaccination. A better understanding of the mechanistic underpinnings of these interactions may have profound implications on the design of cholera prophylaxis and treatment. Here we will focus on just a subset on the rapidly expanding field of how inter-bacterial and host–microbe interactions with commensal microbes influence *V. cholerae* behavior, pathogenesis, and host response to infection (Figure 1).

## The microbiome as a target and driver of cholera infection

### Animal models of *V. cholerae*–microbiota interaction

Tractable animal models for *V. cholerae* are essential in order to define the underlying molecular mechanisms contributing to *in vivo* pathogen fitness and behavior. It was not until 1954 that a successful cholera animal model was developed in infant rabbits, which under certain conditions were highly susceptible to *V. cholerae* infection leading to diarrhea and death, the most relevant clinical outcomes.^14^ The infant rabbit model is still used today to model the next generation of cholera vaccines,^15^ and allows for the study of gross pathological characteristics of diarrhea and death. However, as the small intestinal tissue in infant rabbits is not fully developed, the ligated ileal loop adult rabbit model was developed to examine *Vibrio*-host interactions in developed small intestines with mature immune tissues and with reduced peristalsis, though this requires considerable surgical expertise.^16–18^ The most commonly used *V. cholerae* model of animal colonization and infection is the infant mouse model, which is accessible and offers the advantage of similar virulence gene expression and requirements for colonization compared to humans.^19^ Notably, the infant mouse model was influential in elucidating the essential role of toxin-coregulated pili (TCP) during the pathogenesis of cholera as evidenced by several key studies.^20–23^ Additionally, the suckling mouse model was used to effectively examine morphological changes of *V. cholerae in vivo*^24^ as well as additional colonization factors such as GbpA, which were found to modulate *V. cholerae* attachment to epithelial cells.^25^ Although suckling mice do not display diarrhea, this model remains a very important *in vivo* model system for examining *V. cholerae* colonization factors.^19,26^

Several non-mammalian systems that have recently gained traction for being low-cost, high-throughput, and genetically modifiable are *Drosophila melanogaster, Caenorhabditis elegans*, and zebrafish, *Danio rerio*. The Drosophila model gained relevance when it was shown that flies are susceptible to oral *V. cholerae* infection and die within a day, exhibiting diarrheal symptoms similar to cholera.^27^ *Drosophila* exhibit a relatively simple microbiome, which enables researchers to examine *V. cholerae* infection in the context of host metabolism. Recently, a *Drosophila* study demonstrated that the type VI secretion system (T6SS) reduces epithelial cell repair mechanisms in a microbiota-dependent manner.^28^ As nematodes are natural predators of bacteria, *Caenorhabditis elegans* has also been used as a valuable invertebrate model for *V. cholerae*. Several experiments have shown that two secreted factors of *V. cholerae*, the protease PrtV and the hemolysin HlyA, have protective functions and cause lethality in *C. elegans*.^29,30^ Indeed, the utility of the *C. elegans* model was demonstrated in a high-throughput genomic analysis to study the effects of cytolysin (*hlyA*) on innate immune responses.^31^ Additionally, an elegant study utilizing recombination-based *in vivo* expression technology (RIVET) demonstrated that mannose-sensitive hemagglutinin (MSHA) is necessary to colonize the pharynx of *C. elegans*,^32^ and that a novel cytotoxin named motility associated killing factor (MakA) mediates *C. elegans* killing in a flagellin-dependent manner.^33^ Lastly, as *V. cholerae* naturally resides in an aquatic environment, the zebrafish *Danio rario* represents an attractive potential model for studying cholera pathogenesis. Various strains of *V. cholerae* were shown to successfully colonize the zebrafish small intestine after a natural exposure route of infection and did so independently of TCP and CT.^34^ Moreover, zebrafish display diarrhea as measured by optical density, independent of several cholera accessory toxins including MARTX A, accessory cholera toxin, and zonula occludens toxin.^35^ While more work will need to be done to explore the underlying mechanisms of this model, the zebrafish provides an additional system to study both O1 and non-O1 strains of *V. cholerae*.

An ideal disease model would combine a physiologically and immunologically mature animal with inoculation through the oral route, activation of virulence factors, and subsequent diarrhea. As such, a model does not currently exist, it is necessary to choose the appropriate animal model for the hypotheses being tested. It is also important to note that all these animal systems have very different microbiome structures from that of humans, though mouse systems are much more similar.^36^ As such, the ability to manipulate microbiomes in a targeted fashion is critical to understanding pathogen-commensal interactions in a colonization or infection model. Several studies, reviewed below, have employed both gnotobiotic and antibiotic-treatment techniques to establish human-associated microbes in various animal colonization models, to determine the effects of commensal microbes on *V. cholerae* infection outcome.

#### Cholera effects on the gut microbiome

Until recently, the impact of cholera on the human gut microbiome was much better understood than the role of the gut microbiome on *V. cholerae* infection outcomes. The profuse watery diarrhea associated with cholera has long been associated with changes in commensal microbial populations; culture-dependent studies have shown that cholera leads to a multi-log reduction in non-*Vibrio* bacteria during acute diarrhea compared to convalescent populations.^37^ Recent studies by Hsiao et al. using deep sequencing of fecal 16S ribosomal RNA gene amplicons examined the fecal microbiomes of adult cholera patients in Bangladesh from clinical presentation to 3 months convalescence after the end of diarrhea.^7^ In concordance with culturing studies, the diversity of the gut microbiome during cholera dropped dramatically during acute disease, becoming overwhelmingly dominated by Streptococci, Enterococci, and Proteobacteria. Species more characteristic of healthy human gut microbiomes were detected as very low abundance reservoirs during disease, but over the course of convalescence expanded to reestablish the gut in a manner similar to microbial succession, the ordered process of microbial colonization seen from infancy. Several other culture-independent studies have demonstrated that this transient dysbiosis in microbiome structure seen in cholera can also be caused by malnutrition,^8^ and diarrhea of multiple etiologies including rotavirus and pathogenic *Escherichia coli* infection.^9,38^ These environmental insults can be common in cholera-endemic areas and thus potentially drive a reinforcing cycle of microbiome-dependent vulnerability to infection.

In addition to causing diarrhea that disrupts native gut microbial communities, *V. cholerae* can also directly compete with commensals through the use of contact-dependent killing via the T6SS.^39,40^ T6SS delivers toxin to ‘prey’ cells by puncturing the bacterial membrane using a spike and tube structure that also shares functional homology with the T4 bacteriophage, while T6SS-encoding cells are protected via the production of cognate immune proteins.^41–44^ *In vitro, V. cholerae* is capable of reducing *S. typhimurium* and *E. coli* survival up to 10^5^ fold using T6SS.^45^ *In vivo*, mutations in T6SS have driven colonization defects compared to wild-type in suckling mice,^44,46^ infant rabbits,^44^ and *Drosophila*.^47^ Zhao et al. showed that *V. cholerae* was able to directly attack host commensal *E. coli* in the suckling mouse model of infection; commensal *E. coli* load was lowered by ~300 fold in the wild-type group compared to a *vipA^−^* T6SS mutant.^48^ Interestingly, T6SS-mediated killing of *E. coli* led to an additional upregulation of *tcp* and *ctx* virulence genes during infection compared to mice lacking T6SS target microbes via an as-yet undefined mechanism. Separately from contact-dependent T6SS killing, *V. cholerae* can also use T6SS to increase host gut contractility to expel resident bacterial species, for example the expulsion of *Aeromonas veronii* in a zebrafish colonization model.^49^ Taken together, these findings suggest that *in vivo* T6SS interactions with the microbiota play a complex role in inter-bacterial competition during infection and driving *V. cholerae* fitness in the gut.

#### Gut microbiome structure as a driver of *V. cholerae* susceptibility

Only a limited number of host genetic factors have been associated with susceptibility and resistance to cholera. The ABH blood group antigens found on the surface of numerous cell types, and specifically the O phenotype that expresses an unmodified H antigen, have been associated with increased severity of cholera symptoms. Indeed, the prevalence of O blood group is low in the Ganges River Delta, a historically significant focal center of cholera infection, suggesting that cholera-associated selective pressures may have driven evolutionary changes in human populations.^50^ A growing body of work, however, has focused on the gut microbiome, the co-evolving native microbial community of the gut. Limitations in the ability to define, culture, and manipulate microbial populations in animal systems have stymied detailed molecular characterization of microbe–microbe and microbe–host interactions in the context of infection and colonization. However, a growing body of work, leveraging advances in germ-free animal systems and multi-omic approaches applied to commensal microbial communities, has elucidated several molecular mechanisms underlying the role of human microbiome structure in susceptibility to *V. cholerae* infection.

Hsiao et al. constructed defined microbial communities of cultured isolates, based on the microbiome structure of healthy humans, and established these microbes in germ-free mice,^7^ which then became highly resistant to colonization by *V. cholerae* in contrast to germ-free animals. As microbial colonization was known and controlled, they then identified one microbe commonly found in healthy human populations, *Blautia obeum* that was a dominant contributor of colonization resistance. Direct competition of *B. obeum* and *V. cholerae* reduced colonization of the latter by 2 logs compared to *V. cholerae* in germ-free animals, and targeted exclusion of *B. obeum* from model healthy microbiomes also significantly increased pathogen load in mice. Another set of studies examined the gut microbiomes of household contacts of Bangladeshi cholera patients who subsequently did or did not develop symptomatic disease.^51,52^ Using metagenomic and machine learning approaches, *Blautia, Ruminococcus, Bifidobacterium*, and *Prevotella* species were associated with household contacts that remained uninfected, while *Streptococcus, Prevotella*, and other *Blautia* species were higher in individuals that were subsequently infected. The presence of multiple species within the same genus in both outcome groups, and findings that modulation of *V. cholerae* susceptibility can be driven by specific enzymatic functions (see below) suggest the specificity of microbial species as major drivers of *V. cholerae* pathogenesis in human populations. Midani et al. also experimentally validated the role of *Paracoccus aminovorans*, a Proteobacterium associated with symptomatic cholera, on *V. cholerae* behavior; co-culture with *P. aminovorans* leads to increased *V. cholerae* agglutination and growth *in vitro*.^52^

Alavi et al. have recently confirmed experimentally that human gut microbiome variation drives divergent *V. cholerae* colonization outcomes by colonizing defined model and complete human fecal microbiomes in germ-free mice and suckling animals depleted of native murine microbes using antibiotic treatment.^26,46^ In both animal systems, *V. cholerae* was able to easily colonize microbiomes similar to diarrhea- and malnutrition-disrupted microbial communities dominated by *Streptococcus*, in contrast to microbiomes more similar to healthy Bangladesh gut communities. This effect was not geographically defined, as a 30-fold difference in pathogen colonization was reported when microbiomes from healthy donors from the United States were transplanted into mice and challenged with *V. cholerae*.

Taken together, the emerging data suggest that disruption of the microbiome by other infectious diarrheas or malnutrition may be a risk factor for cholera, but emphasize that modulation of disease susceptibility by commensal microorganisms is not strictly a dichotomous comparison between “normal” and “diseased,” but rather varies on an interpersonal basis, potentially as the result of multiple specific drivers. Importantly, experimental manipulations of the microbiome, where specific microbial taxa can be added or removed, allows for determination of causal effects on colonization/infection outcomes. In human populations, the effects of specific commensal microbes can be confounded by other factors associated with major environmental insults to microbiome structure, for example, diarrhea from different infections, or malnutrition. Some studies have indicated modified immune responses to *V. cholerae* as a function of helminth co-infection.^53^ While malnutrition itself has not been significantly associated with cholera susceptibility,^54^ the numerous other effects of malnutrition on intestinal and immune function may have microbiome-independent effects on infection outcome.^55^

## Commensal microbes and *V. cholerae* virulence regulation during infection

**Figure 2.**
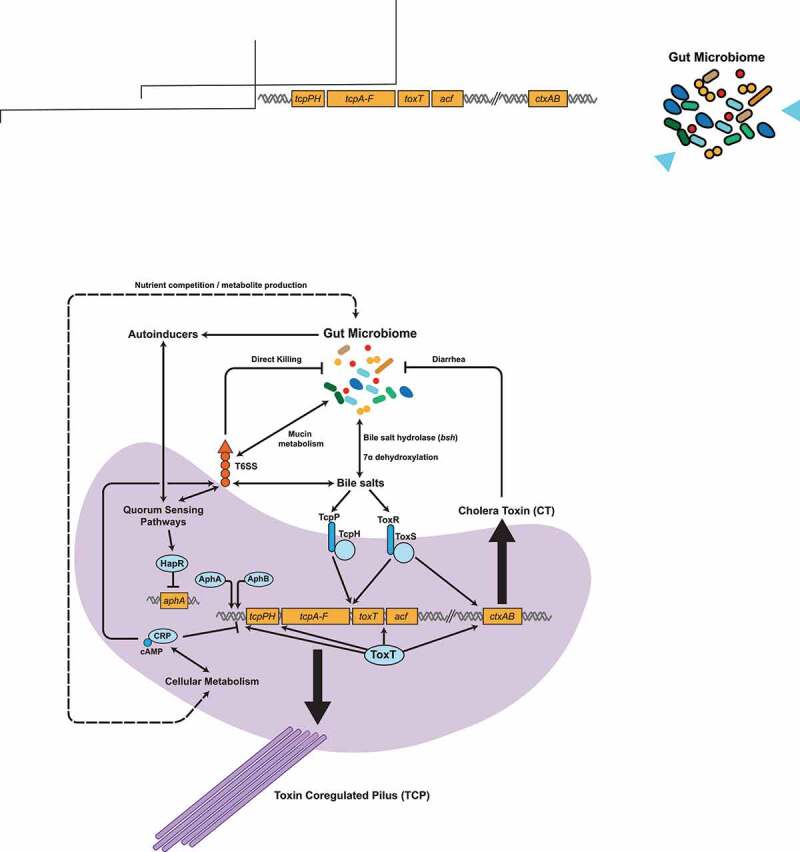
**Virulence gene expression, including that of the key virulence factors cholera toxin (CT) and the toxin coregulated pilus (TCP), can be influenced by numerous pathways controlled by gut commensals**. The gut microbiome is able to produce or modulate several molecular factors leading to changes in *V. cholerae* virulence gene expression, including the production of quorum sensing autoinducers, the metabolism of bile molecules and host environmental nutrients such as mucin

*V. cholerae* is a native organism of aquatic environments such as brackish water and estuaries, often complexed with marine organisms such as zooplankton (Figure 1).^56–59^ In the aquatic reservoir, *V. cholerae* is often found within biofilms that enable attachment to nutritive substrates such as plankton exoskeletons.^58,59^ These biofilm structures also represent an important host infection mechanism^60^ as biofilm-associated *V. cholerae* are much more acid-tolerant than planktonic cells, which are essential for passage through the stomach acid barrier at the beginning of human infection.^61^ Upon transition into the gut, *V. cholerae* undergoes a carefully orchestrated set of gene expression changes in order to adapt to host-specific environmental stresses and cause disease. This transcriptional program is triggered by a series of environmental signals such as temperature, osmolarity, oxygen concentration, and exposure to host-specific molecules such as bile acids, and leads to the elaboration of a number of virulence factors critical to colonization, persistence, and pathology (Figure 2). The two major virulence determinants of *V. cholerae* are Cholera Toxin (CT), which is responsible for the characteristic diarrhea of cholera, and the Toxin-Coregulated Pilus (TCP), which is required for colonization of the intestinal mucosa in both humans and mice.^22,62^ CT is encoded by the *ctxAB* genes on the lysogenic CTXΦ bacteriophage,^63^ and TCP serves both as the receptor for CTXΦ^63,64^ and in microcolony formation at the intestinal epithelium.^65^ TCP biosynthetic genes, including that of the primary structural subunit TcpA and accessory colonization factor (*acf*) genes and several transcriptional activators of virulence gene production, are found on a 40-kb *Vibrio* pathogenicity island.^66^ Both *ctxAB* and *tcpA* are activated by the activity of the AraC/XylS-family transcriptional regulator ToxT,^67–70^ which binds to a degenerate 13-bp DNA sequence known as the ‘toxbox’ in target promoters.^71^ Several factors comprise a complex regulatory path to ToxT expression. ToxR was the first identified positive regulator of *V. cholerae* virulence,^72^ and together with the regulator TcpP activates transcription of *toxT*.^73–75^ The expression of *tcpP* is in turn regulated by the transcriptional regulators AphA and AphB, which cooperatively binds to the *tcpP* promoter, while AphB is able to enhance the *toxR* transcription.^76^

A growing body of work has identified several microbiota-driven factors that influence this tightly controlled program of virulence gene regulation during infection, thus providing a mechanistic basis for the role of microbiome variation in driving divergent outcomes of cholera infection. We will review just a subset of this work, focusing on direct interactions with *V. cholerae*.

### Quorum sensing, the gut microbiome, and *V. cholerae* pathogenesis

**Figure 3.**
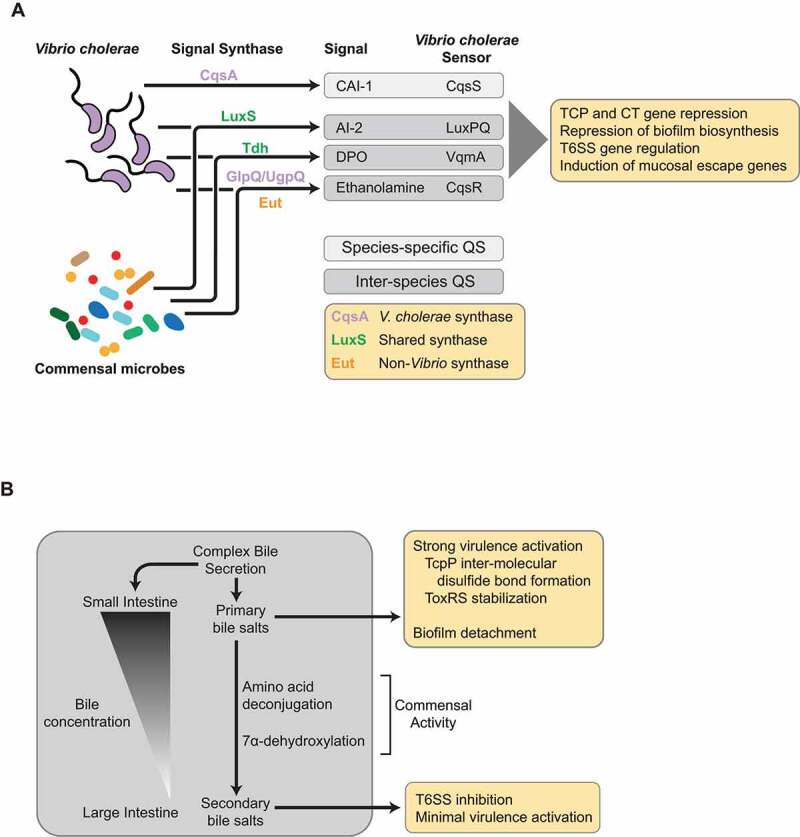
**Production of small signaling molecules by the gut microbiome influences gene expression in** *V. cholerae*. (**A**) Gut commensals are able to interface with *V. cholerae* gene regulation during infection through the production of autoinducer (AI) molecules associated with quorum sensing (QS). QS pathways can be species specific or inter-species, and numerous AI signaling molecule/sensor pairs have been described in *V. cholerae*. Broadly, QS activation by intra- and inter-species specific AI signaling in *V. cholerae* can lead to repression of virulence gene expression, and modulation of pathways associated with inter-bacterial competition (ex. T6SS), and detachment from the mucosa. Thus, premature activation of QS by commensal microbes may disrupt the ability of *V. cholerae* to properly time virulence gene activation during early infection, and affect the outcome of infection *in vivo*. (**B**) The gut microbiome is able to dramatically shape the composition of the bile salts within the preferred site of *V. cholerae* colonization, the distal small intestine. Bile components can be bacteriostatic but also act as a key signal for microbes in the small intestine, and numerous microbes adapted to this environment have enzymatic pathways that alter the structure and activity of bile salts. In *V. cholerae*, primary bile salts promote strong virulence gene activation through interaction with the TcpPH and ToxRS upstream regulatory complexes, and also promotes detachment from biofilms to allow for spread to host tissues. Commensal activity processes primary bile salts to secondary forms, leading to minimal virulence gene activation *in vivo* and inhibition of type VI secretion activity (T6SS)

Quorum sensing (QS) is a bacterial communication system that uses the production and sensing of diffusible signaling autoinducer (AI) molecules to monitor intra- and inter-species population density, allowing coordinated regulation of effector functions by microbial populations (Figures 2, Figure 3a).^77,78^ In *V. cholerae*, QS is capable of repressing the expression of virulence- and biofilm-associated genes at high cell density (HCD), while at low cell density (LCD), such as during early infection, QS is inactive and virulence gene and biofilm biosynthetic gene expression is active. At LCD, the *V. cholerae* QS regulatory system acts to phosphorylate the regulator LuxO via a phosphorelay protein LuxU.^79,80^ Phospho-LuxO is then able to activate the expression of a set of small non-coding regulatory RNAs, Qrr1-4 (quorum regulatory RNAs)^81^ that employ a number of mechanisms to suppress QS gene activation, including the *hapR* gene encoding the master QS regulator HapR, and activate production of the virulence activator AphA.^82^ At HCD, when autoinducer concentrations are high, LuxO becomes de-phosphorylated and Qrrs are not produced, allowing for the expression of HapR. HapR is then able to repress virulence gene expression, via direct repression of *aphA*, as well as the expression of biofilm biosynthetic genes.^81,83,84^

*V. cholerae* cells are able to produce a diverse set of different AI signals that integrate with LuxO as well as other gene regulatory pathways.^85,86^ The *V. cholerae* AI molecule CAI-1 ((S)-3-hydroxytridecan-4-one)^87,88^ is synthesized by the enzyme CqsA.^89^ While long thought to be specific to *Vibrio*s, recent work has demonstrated that pathogenic *E. coli* are also able to sense this autoinducer.^90^ CAI-1 levels are monitored by the membrane-bound histidine kinase sensor CqsS,^91^ which acts as a kinase at low cell- and AI-density, auto phosphorylating and transferring this phosphate to LuxU and thence to the regulator LuxO, leading to Qrr sRNA expression and the upregulation of *aphA* and repression of *hapR*. At high cell density and thus high CAI-1 concentrations, CAI-1 binds to CqsS converting it from kinase to phosphatase activity, leading to the dephosphorylation of LuxO.^79,92^ The consequent loss of Qrr sRNA expression leads to the repression of *aphA* and thus virulence gene expression, as well as the repression of biofilm formation via the activity of HapR.^91,93–95^

Several other autoinducers produced and sensed by *V. cholerae* are inter-species in nature and thus potentially active during infection of host compartments bearing complex microbial communities. One inter-species autoinducer that is broadly distributed amongst gut microbes and that plays a role in virulence gene regulation in *V. cholerae* is autoinducer 2 (AI-2), synthesized by the enzyme LuxS from 4,5-dihydroxy-2,3-pentanedione (DPD). Homologs of *luxS* are found in *V. cholerae* (*VC0557*) as well as the genomes of more than 500 Gram-positive and Gram-negative bacterial species.^77^ In *V. cholerae*, AI-2 is sensed through the LuxP/Q signaling pathway.^96^ LuxP is located in the periplasm and forms a heterotetramer when joined with LuxQ. At LCD when AI-2 is not bound, LuxQ acts as a kinase and auto-phosphorylates the cytoplasmic domains, leading to the phosphorylation of LuxU and then LuxO. At HCD, the binding of AI-2 facilitates a conformational change, breaking the symmetry of the LuxPQ heterotetramer, thus interrupting the phosphorylation cascade and leading to repression of virulence factor expression.^97^ Several different active structures of AI-2 have been identified. In *Vibrio*s, AI-2s are produced as a furanosyl borate diester compound ((2S,4S)-2-methyl-2,3,3,4-tetrahydroxytetrahydrofuran borate),^87,98^ in contrast to the cyclized but non-borated DPD derivative found in *E. coli* and *Salmonella* spp.^99^ The interspecies nature of these AI-2 molecules is highlighted by bacteria that lack *luxS*, for example *Pseudomonas aeruginosa*, is still capable of detecting AI-2 produced by other bacterial species and accordingly altering gene expression.^100^ Similarly, though able to produce their own AI-2, *V. cholerae* cells can sense other AI-2 forms, as cell-free supernatants of *E. coli* are able to induce gene expression changes in *Vibrio*s depending on the ability of *E. coli* to produce AI-2.^101^

Direct signaling may also occur at cross-kingdom levels between mammalian host and the pathogen. Some bacterial growth and virulence expression respond to mammalian stress hormones, including *E. coli* and other gram-negative bacteria. For example, the neuroendocrine stress hormone norepinephrine is capable of stimulating cellular proliferation in *E. coli*;^102,103^ addition of norepinephrine to growth media increased the growth rate of *E. coli* O157:H7 by several logs, with a 100–160 fold increase in toxin production during the first 12 hours of culture time.^104^ The Bassler lab demonstrated that mammalian epithelial cells from colon, lung, and cervical tissues are capable of producing an AI-2 mimic that can be detected by bacterial AI-2 receptors in *Vibrio harveyi* and *Salmonella typhimurium*, suggesting that host-produced molecules may be capable of interfacing with gut microbial QS regulation.^105^

In addition to the CAI-1/AI-2 QS pathways acting through LuxO/HapR described above, several novel signaling molecules and QS receptors have recently been identified. The intestinal metabolite ethanolamine has been shown to regulate *hapR* expression through the regulator CqsR.^85,106,107^ Ethanolamine is sensed by the CqsR periplasmic CACHE ligand binding domain with high specificity, and addition of ethanolamine repressed Qrr sRNA expression and increased *hapR* expression leading to inhibition of colonization of the mouse small intestine.^106^ Levels of ethanolamine may be controlled and sensed by numerous bacterial pathways. For instance, in pathogens such as enterohaemorrhagic *E. coli* (EHEC), ethanolamine has been shown to increase virulence gene expression.^108^ Similarly, ethanolamine metabolism and Type III secretion system are regulated by environmental ethanolamine levels in *Salmonella*,^109,110^ and other common gut pathogens such as *Enterococcus faecalis*^111^ and *Clostridioides difficile*^112^ also exhibit ethanolamine-dependent gene regulation. Papenfort et al. recently demonstrated that 3,5-dimethylpyrazin-2-ol (DPO) acts as a QS signaling molecule in *V. cholerae*.^86^ DPO is synthesized from threonine and alanine by the enzyme threonine dehydrogenase (Tdh); threonine metabolism is commonly observed in several intestinal microbes including *E. coli*.^113^ DPO is able to bind to the LuxR family transcriptional regulator VqmA, and in so doing leads to the increase in the transcription of the small regulatory RNA VqmR, leading to the downregulation of accessory toxin genes and the *vps* genes involved in biofilm synthesis. Expression of VqmA was previously shown to be deleterious to *V. cholerae* infection and virulence expression via direct activation of HapR without modification of DPO synthesis.^114^

Recently, Hai Wu et al. have solved the crystal structure of the VqmA-DPO-DNA complex, demonstrating a direct interaction between DPO and the PAS ligand binding domain of VqmA, and speculated that DPO and DNA binding may stabilize VqmA.^115^ In another study, they observed conformational differences when VqmA is not bound to the target promoter DNA, leading to a possible AI-dependent regulation differential mechanism.^116^ Additional work by Mashruwala et al. suggested VqmA activity is related to cell density, environmental oxygen levels, and host produced bile.^117^ In the microaerophilic gut environment, CAI-1 and AI-2 production increased, and VqmA was shown to form disulfide bonds leading to increased transcriptional activity. The presence of bile salts disrupted these disulfide bonds, leading to an observed increase of *tcpA* and biofilm-associated *vps* gene expression. The inter-species nature of DPO and VqmA in the gut is highlighted by recent studies showing that a *Vibrio parahemolyticus-*bacteriophage-encoded VqmA is able to respond to DPO in the gut and mediates cell lysis by activating expression of the phage gene *qtip*; Qtip sequesters the phage cl repressor and leads to bacterial host lysis.^118^

The diversity of different interspecies signaling molecules produced by commensal microorganisms underlines the complexity of the QS environment of the gut during infection. Several studies have examined the ability of targeted manipulation of QS to affect both gut microbiome structure and outcomes of *V. cholerae* colonization and infection. Experiments conducted by Thompson et al. show that by modifying the Lsr AI-2 transport pathway, transgenic *E. coli* could alter intestinal AI-2 levels in antibiotic-treated mice, leading to an AI-2 dependent difference in relative abundance between two major bacterial phyla of gut commensals, the Bacteroidetes and Firmicutes.^119,120^ Duan et al. employed *E. coli* Nissle 1917 as a carrier to express CAI-1 via expression of *cqsA*.^121^ They found that pretreating suckling mice with CAI-1-producing *E. coli* for 8 hours could increase mouse survival by over 90% upon infection with *V. cholerae*, and that co-ingestion of CAI-1-producing *E. coli* and *V. cholerae* resulted in a 25% increase in survival rate post-infection.

QS-mediated interference in *V. cholerae* pathogenesis is not restricted to artificial manipulation. Studies have shown that the common human gut commensal *Blautia obeum* encodes a functional AI-2 synthase *luxS* that drives reduced *tcpA* expression in *V. cholerae* and mediates microbiome-mediated resistance to infection.^7^ In germ-free mice inoculated with both *B. obeum* and *V. cholerae*, expression of *luxS* in *B. obeum* increased and *V. cholerae* colonization was ablated. Targeted removal of *B. obeum* from defined microbial communities established in germ-free animals dramatically reduced the ability of these microbial assemblages to resist invasion by *V. cholerae*, and transgenic expression of the *B. obeum luxS* in AI2-*E. coli* was sufficient to restrict *V. cholerae* colonization in gnotobiotic mice. This signaling was independent of the canonical LuxP AI-2 sensor system; deletion of *luxP* did not rescue the ability of *V. cholerae* to colonize when *B. obeum* was present in the gut. However, expression of *vqmA* was increased during infection in response to *B. obeum*, and *V. cholerae* lacking *vqmA* showed improved colonization in the presence of *B. obeum* compared to wild-type pathogen. Interestingly, *hapR* expression did not seem to respond strongly to increased *vqmA*. Taken together with findings of DPO interaction, these data suggest that this multi-functional AI-sensor/regulator may respond to several QS signaling pathways with different regulatory targets. The dispensability of LuxP to signaling with *B. obeum* AI-2 also suggests that there may be substantial un-characterized diversity in the structure and function of these inter-species autoinducers.

These recent advances in studying cross-species QS signaling in the gut, and the ability of these pathways to interfere with key *V. cholerae* infectious processes such as TCP biogenesis and biofilm production suggest that further characterization of QS in the microbiome may yield novel clinical therapeutic and prophylactic targets for cholera management.

#### Microbiome-driven modification of the gut chemical environment controls *V. cholerae* gene expression

In addition to QS systems, the action of commensal microbes can affect the levels and function of several other components used by *V. cholerae* to appropriately time virulence gene activity in the gut. One of these major virulence-regulatory components is bile, a digestive secretion that aids in emulsification and solubilization of dietary lipids. Bile is a complex mixture of compounds comprising bile acids, cholesterol, phospholipids, and immunoglobulins.^122^ The synthesis of the predominant bile component, bile acids, occurs in the liver from cholesterol, often in amino-acid conjugated forms containing taurine and glycine. Bile is stored in the gall bladder and secreted into the small intestine in response to food intake. The local pH of the intestine means that bile acids are often found as primary bile salts and can be further modified by the action of gut bacteria into secondary forms.^123–125^ Up to 95% of secreted bile acids are reabsorbed within the distal ileum and passed via portal circulation back to the liver to be re-conjugated to amino acids and re-secreted.^126,127^

The detergent nature of bile salts and the activities of the various other bile components can have potent bacteriostatic activity, affecting membrane stability and cellular homeostasis; pathogenic and commensal gut microbes have evolved mechanisms to survive and exploit this gut-specific component.^128^ *V. cholerae* bile resistance is mediated by the action of efflux pumps and outer membrane porins in bile salt accessibility to the cell.^129–132^ Since bile secretion and re-absorption predominantly occurs in the small intestine, the favored site *of V. cholerae* colonization, this pathogen has evolved mechanisms to take advantage of this intestinal-specific signal in order to time expression of virulence genes (Figure 3b). A set of primary bile salts (e.g. taurocholate, glycocholate) has been shown to activate expression of virulence genes by affecting the structure and function of several key transcriptional regulators of virulence. Taurocholate has been shown to increase TcpP activity by promoting the formation of intermolecular disulfide bond formation and dimerization under microaerophilic/reducing conditions,^133^ and bile salt-induced TcpP–TcpP interactions are further enhanced by the presence of calcium.^134^ Bile salts have also been shown to modulate ToxR activity by preventing proteolysis of ToxR and promoting formation of ToxRS complexes.^135,136^ Taurocholate has also been shown to promote detachment from biofilm structures, enabling *V. cholerae* to colonize mucosal surfaces after passage through the gastric acid barrier.^137^ The heterogenous nature of bile means that other bile components have been shown to drive variable effects on *V. cholerae* virulence. For example, unsaturated fatty acids in bile and crude bile can inhibit the transcriptional activity of ToxT,^138^ and a mixture of bile salts can also reduce the ability of VqmA to mediate QS-dependent repression regulation of biofilm and virulence.^117^

One key mechanism for commensal gut microbes to control the bacteriostatic activity of bile is the expression of bile salt hydrolase (*bsh*) enzymes, which mediate the hydrolysis of amino-acid conjugated bile salts and reduce the detergent-like effects of bile and increase bile salt resistance.^139–141^ The importance of microbial activity in modulating bile composition in the gut can be seen in germ-free mice, where essentially all bile acids in the small intestine are amino acid conjugated, in contrast to conventionally reared animals.^142^ Bioinformatics analyses show that BSH is broadly distributed among members of the human gut microbiota and can be classed into several broad phylotypes that differ in substrate specificity and activity.^125^ Thus, the presence and expression of different microbial enzymes can have dramatic effects on the bile acid pool of the intestines, with consequent differential effects on *V. cholerae* gene regulation and responses in these different microbiome contexts.

Recent work by Alavi et al. has demonstrated that the *bsh* activity of the *V. cholerae*-restricting commensal microbe *B. obeum* is able to contribute to *V. cholerae* infection outcomes.^46^ This work demonstrated that *B. obeum* encodes a *bsh* with high activity against the key virulence-activating factor taurocholate. The presence and activity of *bsh* was shown to be higher in healthy human gut microbiomes *in vitro*, and the fecal metagenomes of healthy Bangladeshi adults were also characterized by higher levels of *bsh* compared to *V. cholerae*-susceptible dysbiotic microbiomes. They demonstrated that this enzymatic activity was able to ablate the induction of *tcpA* expression in response to intestinal tissues through depletion of taurocholate levels, and that *in vivo*, the presence of *B. obeum bsh* activity was associated with lower *tcpA* expression and *V. cholerae* colonization. These effects were independent of AI-2, as this commensal-encoded enzyme was able to ablate *tcpA* activation by intestinal tissues even when these tissues were boiled to remove AI-2. Expression of *B. obeum bsh* by a natively *bsh^−^ luxS^−^ E. coli* was also able to significantly reduce *V. cholerae* colonization in suckling mice. These data suggest that differential capacity for bile metabolism by commensal microbes is a key driver of individual- and microbiome-specific differences in *V. cholerae* infection outcome and may serve as a recurrent window of vulnerability to infection by *V. cholerae*.

A combination of microbiota-driven effects on chemical signals in the gut may also be important for the regulation of T6SS-mediated pathogen-commensal competition. Several studies have demonstrated a link between QS and regulation of T6SS; HapR directly regulates T6SS genes^143^ and indirectly through the action of QstR,^144^ and QS sRNAs can repress T6SS-related gene expression.^95^ T6SS regulation is also affected by several processes that intersect with the functions of the microbiota. Bile acids are also able to regulate T6SS gene expression, with deoxycholic acid, a secondary bile acid generated via microbial 7-α-dehydroxylation of cholic acid, shown to inhibit assembly of the T6SS apparatus.^145^ Components of mucus, the protective glycoprotein coat at the intestinal mucosa, are able to de-repress T6SS gene expression.^145^ Since numerous commensal microbes have been shown to metabolize mucus in the gut environment (see below), and the role of microbes in bile metabolism has been intensively investigated, complex microbiota-driven mechanisms may thus serve as triggers for the control of inter-microbial killing mechanisms during infection.

### Diet and nutrient acquisition drive the composition of the gut microbiota and *V. cholerae* infection

Environmental factors, especially diet, play a dominant role in shaping the human gut microbiota.^146^ Long-term diet shapes the composition of the gut microbiota: high protein and high fat diet (western diet) lead to a *Bacteroides* dominated enterotype, while the high carbohydrate diet yields a *Prevotella* dominated enterotype.^147^ Short-term dietary intervention of shifting macronutrients is also able to dramatically alter the structure and function of the gut microbiome in several human studies.^148,149^ For example, a high fat diet caused an increased secretion of bile acids, enriching for bile resistant microbial taxa such as *Bilophila wadsworthia, Alistipes putredinis*, and *Bacteroides sp*., and the expression of bacterial genes encoding bile salt hydrolases, compared to a plant-based high fiber diet.^149^ Some studies have used targeted dietary manipulation to drive the expansion of specific taxa within the gut microbiome,^150^ but despite the importance of understanding dietary contributions to microbiome structure and *V. cholerae* metabolism during infection, this area of research remains comparatively underdeveloped.

#### Central metabolism and virulence

*V. cholerae* has evolved the ability to vary the regulation of a variety of virulence and metabolic genes to better colonize and compete with resident gut microbes. While *V. cholerae* is able to rapidly grow to high cell density during infection, the nutritional requirements of this pathogen *in vivo* have not been well defined. Several studies have shown that central metabolism affects colonization and virulence gene regulation. Deletion of *edd*, which encodes 6-phosphogluconate dehydratase in the Entner-Doudoroff (ED) pathway for sugar catabolism, causes the decreased expressions of virulence genes *ctxA* and *tcpA*, and the regulator *toxT*, as well as diminished colonization in suckling mice and reduced fluid accumulation in a ligated rabbit ileal loop model.^151^ Activation of the ED pathway can inhibit biofilm formation *in vitro*.^151^ Gluconeogenesis, which converts the non-carbohydrate precursors to glucose, also affects *V. cholerae* pathogenesis. The phosphoenolpyruvate synthase (PpsA) converts pyruvate into phosphoenolpyruvate (PEP), and the phosphoenolpyruvate carboxykinase (PckA) converts oxaloacetate into PEP. Deletions of the *ppsA* and *pckA* genes resulted in decreased *V. cholerae* colonization in both adult and infant mouse models, and decreased motility and biofilm formation.^152^ This may be especially important in the context of competition with members of the gut microbiota, as colonization defects in these mutants were worsened by approximately ten-fold in the presence of commensal microbes. *In vitro*, manipulation of TCA by inhibition or supplementation with citrate has been shown to increase *toxT* expression and acetate secretion under aerobic conditions.^153^ *In vivo* studies demonstrated *V. cholerae* can use citrate as a carbon source during infection and to improve fitness in the presence of commensal microbes. Supplementation of citrate in an adult mouse colonization model led to a loss of fitness of a citrate fermentation mutant (Δ*citAB*) compared to wild-type in the presence of gut microbes, and promoted microbial growth in general in the small intestine.^154^ The role of general nutrient availability is also highlighted by several studies focusing on the role of cyclic AMP (cAMP) receptor protein (CRP) on the regulation of various virulence-associated pathways in *V. cholerae*. In the absence of preferred nutrient sources, possibly via inter-microbial competition *in vivo*, CRP-cAMP is able to activate components of the T6SS.^155^ The cAMP-CRP complex also negatively regulates the expression of CT and TCP,^72^ and directly binds and negatively regulates the promoter of the virulence activator genes *tcpPH*.^156^ Chromatin immunoprecipitation mapping of CRP binding sites suggests a substantial overlap of CRP-regulated genes and the ToxR regulon.^157^ These findings suggest that complex commensal microbial communities *in vivo* are able to modulate nutrient pools available for *V. cholerae*, which may have profound influences on the regulation of virulence associated factors during infection.

#### Alternative electron acceptors

**Figure 4.**
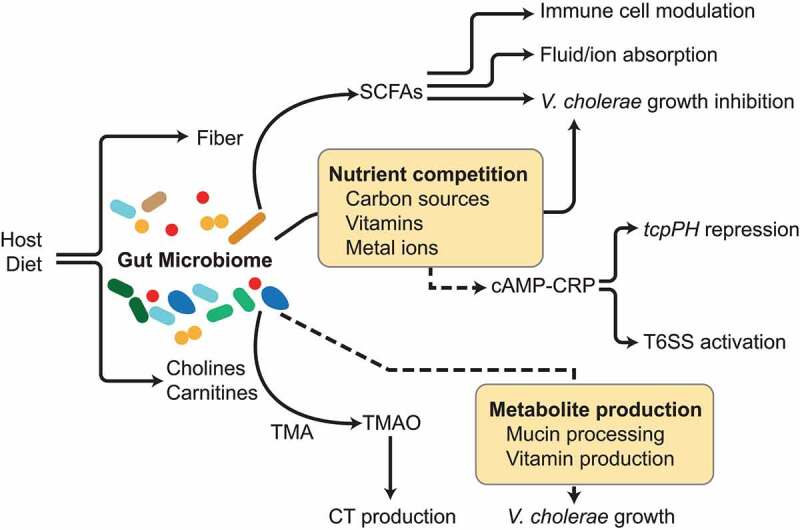
**Production of and competition for nutrients between intestinal microbes can influence *V. cholerae* growth and pathogenesis**. Commensal microbes can produce short-chain fatty acids (SCFAs) from dietary fiber, which subsequently influence host and microbial behavior, including the activity of immune cell subtypes, fluid and ion balance and the intestinal gut barrier, and direct inhibition of *V. cholerae* growth. Nutrient competition between commensals and *V. cholerae* can modulate *V. cholerae* growth but also virulence gene regulation. For example, microbes can process dietary cholines and carnitines to drive production of trimethylamine oxide (TMAO), which can act as an alternative electron acceptor that promotes anaerobic *V. cholerae* growth and cholera toxin expression. Microbial competition for nutrients such as carbon sources can also lead to modulation of virulence gene expression from cAMP-CRP

*V. cholerae* is a facultative anaerobic bacterium, capable of adapting to fluctuating oxygen levels. *V. cholerae in vivo* expansion is driven largely by aerobic metabolism, consistent with observations that *V. cholerae* preferentially replicates within the epithelial crypt spaces with greater oxygenation that enables oxidative metabolic pathways.^158^ *V. cholerae* uses pyruvate dehydrogenase (PDH) to expand in the small intestine, rather than pyruvate formate-lyase (PFL) mediated anaerobic metabolism to convert pyruvate to acetyl coenzyme A (acetyl-CoA), which provides growth support during infection.^159^ Moreover, the cholera toxin (CTX)-induced increase of cAMP can induce host cells to switch to anaerobic respiration, leading to host reduced consumption of oxygen.^160^ However, under hypoxic conditions, *V. cholerae* is able to employ several alternative electron acceptors (AEA) such as fumarate, trimethylamine N-oxide (TMAO), and NO_3_^－^.^161^ Many enteric pathogens contain the NO_3_^－^ reduction pathway that can convert NO_3_^－^ to N_2_ gas or NH_4_^+^,^162^ but *V. cholerae* contains only the nitrate reductase Nap and lacks the downstream reductases,^163^ leading to NO_2_^－^ accumulation that can inhibit glycolysis. However, *V. cholerae* is capable of undergoing NO_3_^－^ respiration using pH-dependent responses. Under alkaline conditions typically found in the small intestine, *V. cholerae* can reduce nitrate to support growth, and NO_3_^－^ respiration may play an integral role in interspecies competition against commensal organisms.^164^ Under low pH co-culture conditions *in vitro* where *V. cholerae* NO_3_^－^ respiration is inactive, *V. cholerae* was outcompeted by *E. coli* K12, which retains NO_3_^－^ respiration activity at this pH, while under alkaline hypoxic conditions where *V. cholerae* NO_3_^－^ respiration is intact, the pathogen efficiently competed with *E. coli*. The small molecule TMAO has been shown to have a positive correlation with increased risk of cardiovascular disease^165^ and metabolic syndrome,^166^ and can be produced by the metabolism of commensal microbes. Gut microbes can affect TMAO levels through the fermentation of choline or L-carnitine to the intermediate compound trimethylamine (TMA), followed by oxidization of TMA by flavin monooxygenases to TMAO in the liver.^167^ The addition of TMAO to an infant mouse model of *V. cholerae* infection promoted CT production during colonization, a process that accelerated in the presence of reactive oxygen species (Figure 4).^168^ Since several commensal gut microbes have been implicated in the accumulation of TMAO,^169,170^ the differential ability of different microbiomes to affect TMAO levels may thus be a metabolic factor in promoting or inhibiting *V. cholerae* pathogenesis.

#### Short-chain fatty acids

Short-chain fatty acids (SCFAs), produced by the gut microbiota fermenting non-digestible dietary fibers and resistant starch, have been highlighted as key-signaling molecules that connect gut microbiome and host health, including inflammatory bowel diseases, diabetes, cardiovascular disease, as well as pathogen resistance.^148,171–173^ SCFAs play a marked role in maintenance of gut barrier function, immune homeostasis, anti-inflammatory effects, and also act as energy sources for epithelial cells.^171^ Anaerobic fermentation of dietary fibers by the gut microbiota produces acetate, propionate and butyrate, which represent 90–95% of the SCFAs in the intestine. The animal-based diet results in a higher concentration of the branched chain fatty acids, such as isovalerate and isobutyrate, which are mainly derived from the amino acids valine, leucine, and isoleucine.^149,174^ The production of SCFAs by the gut microbiota may benefit cholera treatment via improvement of sodium and water absorption, and the promotion of host immune response to cholera toxin (Figure 4).^175^ The application of the unabsorbed carbohydrates that can be fermented by gut microbes has also been shown to improve cholera management: addition of maize starch resistant to digestion but able to be fermented to the standard glucose-based oral rehydration therapy reduced the fecal fluid loss and shortened the duration of diarrhea in one human study.^176^ Using a germ-free mouse model with gut microbiota derived from undernourished Bangladeshi children, Di Luccia et al. conducted a combined prebiotic and probiotic intervention that successfully improved mucosal IgA responses to cholera toxin, associated with the level of SCFAs.^177^ Another recent study with murine commensal microbes demonstrated that antibiotic treatment of animals led to a depletion of colonization-resistant taxa such as *Bacteroides vulgatus* and *V. cholerae*-inhibitory microbiota-derived SCFA metabolites, and an ablation of mucus catabolism.^178^

#### Nutrient exchange and competition with commensal microbes

To adapt to colonizing in a mammalian environment, the gut microbiota evolved characteristics to maximize access to food. When pathogens invade the nutrient-limiting intestinal habitat, they have to compete with the predominant residents for resources (Figure 4). One example is the competition for amino acids, an essential macronutrient. The enteric pathogen *Citrobacter rodentium* is forced to activate amino acid biosynthesis pathways to survive in conventionally raised mice, but not in germ-free or antibiotic-treated animals, highlighting the importance of nutrient-competition and cross-feeding between pathogens and commensal microbes.^179^ At the intestinal mucosa, the thick layer of mucus glycoprotein serves as a barrier to reduce bacterial access to epithelial cells, but also as an important nutrient source for gut microbes. Mucus can serve as a carbohydrate reservoir, providing a variety of carbohydrate nutrients to bacteria, such as *N*-acetylgalactosamine (GalNAc), *N*-acetylglucosamine (GlcNAc), galactose, fucose, and sialic acid (*N*-acetylneuraminic acid, Neu5Ac).^180^ Mucin degradation is also associated with the pathogenicity of enteric invaders.^159^ Toxigenic strains of *V. cholerae* contain the *nan* cluster on the pathogenicity island VPI2, which allows the use of sialic acid as a carbon source.^181^ Inactivation of sialic acid utilization caused a decreased colonization of *V. cholerae* in an infant mouse model,^182^ while the mucus components GlcNAc and NeuAc promote *V. cholerae* motility.^183^ Genes involved in the metabolism of those mucin glycans can be found in gut microbial commensals, such as species from *Bacteroides, Lactobacillus, Bifidobacterium*, and *Akkermansia muciniphila*.^159,184,185^ Thus, the ability of different assemblages of commensal bacteria to metabolize mucin may be an important driver of *V. cholerae* fitness in the gut. Prior work highlights the potential for this interaction; the presence of mucin glycan utilizers in the gut such as *A. muciniphila, Bacteroides intestinihominis*, and the generalist carbohydrate utilizers *Bacteroides thetaiotaomicron*, and *Bacteroides caccae* can increase *C. rodentium* susceptibility due to the enhanced mucus degradation,^186^ while other studies have used consortia of commensal metabolizes of mucus components to deny these resources to *C. difficile* and thus inhibit colonization.^187^

Vitamins as micronutrients have important effects on host immunity, and act as important regulators of growth, differentiation, and proliferation of epithelial cells directly on the host as well as through regulating the composition of the gut microbiota.^188–190^ Notably, it has been shown that vitamin deficiency increases the risk of infection.^191^ In one study, vitamin A-deficient rats had a reduced response to an oral cholera vaccine, due to the decreased number of the IgA-producing cells in the mesenteric lymph nodes, which resulted in lower concentrations of total IgA, as well as specific anti-CT IgA antibody levels.^188^ *L*-ascorbate (Vitamin C) can be used as an alternative carbon and energy source for *V. cholerae*, and the inability to utilize *L*-ascorbate causes competitive defects in *in vitro* growth in M9 minimal media supplemented with casamino acids and intestinal mucus, suggesting that *L*-ascorbate fermentation may play a role in an *in vivo* phenotype.^192^ Humans lack the ability to biosynthesize many vitamins, relying on dietary intake and the metabolic activity of commensal microbes. The commensal microbes of the gut microbiome have been shown to produce several vitamins, including vitamin K and water-soluble B-vitamins such as cobalamin and folates.^193^ The cobalt-containing corrinoid vitamin B_12_ is exclusively derived from microorganisms, especially anaerobic commensals.^194^ A comparative genomic analysis of 11,000 bacterial species showed that 86% of bacteria contain B_12_-related processes, but only one-third are able to carry out *de novo* synthesis of B_12_;^195^ most bacteria rely on transport mechanisms to obtain it.^196^ For instance, microbes develop elaborate mechanisms to salvage corrinoids, and one good example is the human symbiont *Bacteroides thetaiotaomicron*, which possess three functional, homologous vitamin B_12_ transporters for distinct B_12_ analogs.^197^ This gene redundancy confers a competitive advantage in a nutritionally limited environment, which can be an effective defense against colonization of pathogens. *V. cholerae* also lacks *de novo* synthesis genes for vitamin B_12_ and needs to compete with gut microbial residents to import the intermediate corrinoids to participate in central metabolism, as in the cobamide-dependent methionine synthase MetH.^198^ While deletion of corrinoid uptake genes did not show a colonization defect^199^ in suckling animals, the effects of these nutrient acquisition pathways in the context of complete human microbiomes have not been well studied. Folate biosynthetic genes are also ubiquitous in reference genomes of gut commensal isolates.^200^ Microbiota-dependent synthesis of folate may also play a role in *V. cholerae* pathogenesis, as folate-like molecules have been shown to regulate *V. cholerae* virulence through interaction with the dinucleotide cyclase DncV (*VC0179*), which is involved in the production of secondary messenger cyclic dinucleotides involved in the regulation of phospholipases that may play a role in pathogenesis, including via modulation of ethanolamine levels.^201,202^

Vertebrates have also evolved mechanisms to tightly regulate metal levels that either restrict access to the nutrient metals or direct excess metals that can be toxic to the enteric pathogens, known as nutritional immunity.^203^ Trace metals are essential for approximately one-third of proteins, acting either as the cofactor or as a prosthetic group for essential enzymes.^204^ Those essential metals, such as Fe, Zn, Mn and Cu are required for bacterial pathogens to invade the host.^203^ Competition for these metal ions is an important factor in the invasion and colonization of complex microbial environments by gut pathogens. Deletion of zinc utilization genes or zinc-regulated genes limits *V. cholerae* growth and also causes colonization defects, and these *in vivo* effects are greatly exacerbated in the presence of the gut microbiota during colonization of adult mice.^205^ Calcium does not affect virulence directly, rather it enhances bile salt-dependent virulence activation through modulating the dimerization of TcpP, a membrane-bound regulator of virulence gene expression.^134^ Most Fe *in vivo* is complexed with heme as a cofactor in the oxygen transport protein hemoglobin. CTX can induce the congestion of the capillaries in the ileum with red blood cells and releases free heme, promoting iron accessibility for *V. cholerae*.^206^ Iron supplementation can also alter gut microbiome composition, stimulating the growth of enteropathogenic bacteria, and reducing the abundance of beneficial commensals from genus Lactobacillus and Bifidobacterium, thus increasing the risk of diarrhea.^207^ As further improvements in experimental models of microbiome–pathogen interactions are made, we can expect additional mechanistic studies on macro- and micronutrient competition during infection, and a further delineation of the critical limiting nutrients for *V. cholerae* growth and population expansion *in vivo.*

### The microbiome in host immune responses to *V. cholerae* infection and oral cholera vaccines

**Figure 5.**
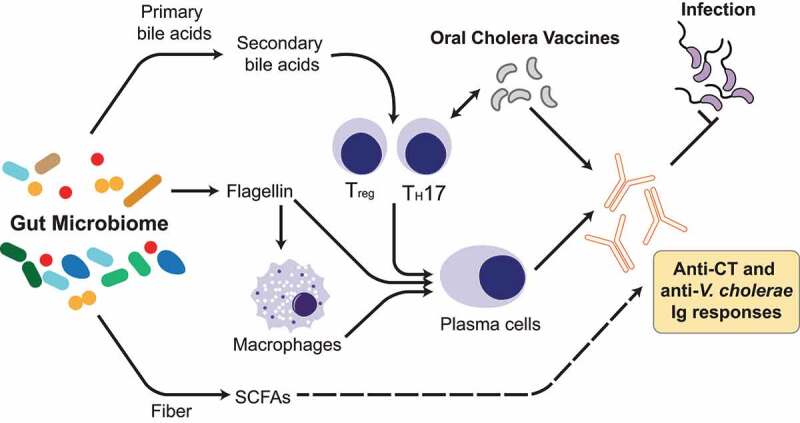
**Interactions of the gut microbiome, host immunity, and *V. cholerae***. Production of specific metabolites by commensal microbes can influence the activity and abundance of specific cell subtypes in the host immune system, including effector and regulatory lymphocytes, macrophages, and plasma cells. These pathways include the production of secondary bile acids and short-chain fatty acids (SCFAs) from dietary fiber. Additionally, bacterial components such as flagellin directly stimulate both macrophages and plasma cells, allowing for a stronger antibody response. Together, these commensal metabolic activities lead to modulation of antibody responses against *V. cholerae* induced by infection and oral cholera vaccines

Vaccination is a key preventative strategy for improving health worldwide, and a critical consideration in the success of vaccination strategies and design is ensuring uniformly high immunogenicity and efficacy. However, several studies have demonstrated differences in immune responses to both infection and oral cholera vaccines (OCVs) between different human populations. A recent clinical study compared serological correlates of protection in age-matched North America and Bangladeshi adults who were voluntarily infected with *V. cholerae* O1 Inaba El Tor N16961. In general, anti-serum antibody responses were predominantly anti-OSP IgA and IgM. Notably, at most timepoints serum anti-CtxB IgA and IgM responses were greater in the naïve North American population than the Bangladeshi participants.^208^ Vaccines developed for gastrointestinal infections such as cholera and rotavirus exhibit less than favorable responses in developing populations as compared to industrialized countries, particularly in children and elderly populations. Several hypotheses for this variation include maternal IgA protection from breast milk,^209^ small bowel overgrowth,^210^ and blood type.^211,212^ Another key difference in the gut environments of individuals in different geographical contexts is the composition of the gut microbiome,^8,11,213^ whose important immunomodulatory functions, including in vaccine response, are supported by a large and growing body of literature (Figure 5).^177,214–220^

#### Oral cholera vaccines (OCVs)

Both killed and live oral cholera vaccines have performed less well in populations of developing countries as compared to developed countries, particularly in younger children. The first licensed killed WC oral vaccine was Dukoral in 1991. It consists of recombinant cholera toxin subunit B (CTB) and three strains of inactivated O1 Classical and one strain of O1 El Tor. It showed a 50% efficacy against all age groups in a large field trial in Bangladesh,^221^ while it resulted in significant vibriocidal titers in 89% of volunteers in the US.^222^ Another study compared the vibriocidal responses in age-matched Swedish children and although they had a lower baseline titer, there was a greater vibriocidal response.^223^ Another second widely approved WCK vaccine, Shanchol, consists of a mixture of serotype O139 and O1 strains without rCTB, and is given in a two-dose regimen over 2 weeks. Shanchol has demonstrated safety and immunogenicity in children and adults^224^ but a broad range of 37%-69% efficacy^225^ across several studies in cholera-endemic areas. Live attenuated cholera vaccines have also been developed and are considered to be more immunogenic and generate stronger mucosal vaccine responses as compared to WCK vaccines.^226^ A live attenuated vaccine strain (CVD103-HgR, manufactured as Vaxchora) has been developed, consisting of the *V. cholerae* O1 classical Inaba strain with 94% of the *ctxA* gene encoding for the enzymatic subunit of CT deleted, and with a mercury resistance cassette inserted into the gene for hemolysin to aid in identification.^227^ A single oral dose was found to be safe and immunogenic, with up to 91% protective efficacy, in US and Swiss volunteers,^228–230^ but exhibited an overall efficacy of 55% in a large-scale field trial in a cholera endemic area.^231,232^ Development of OCVs is continuing; another promising live-attenuated vaccine is CholeraGarde, consisting of O1 El Tor Inaba with CT deleted. This was safe and immunogenic in US and Bangladeshi adults as well as Bangladeshi toddlers and infants, albeit with lower cholera toxin responses as seen in Bangladesh adults.^233–235^ Most recently, the Waldor group has developed a live attenuated cholera vaccine based on the wild-type Haiti outbreak strain.^15^ The live-attenuated strain, HaitiV, has nine modifications that render it to be less virulent but still impart long-term immunity. Their results indicate that HaitiV mediates colonization resistance to wild type *V. cholerae* when given 24 hours prior to wild-type infection. While long-term protection was not measured, the vaccine exhibits probiotic-like protection,^15^ and HaitiV elicits strong correlates of protection in offspring of immunized dams, independent of HaitiV colonization.^236,237^

#### Microbiota–vaccine interactions

While an expanding body of work has demonstrated that microbiome structure is able to drive various aspects of *V. cholerae* pathogenesis and *in vivo* fitness, the majority of studies demonstrating a microbiota link to vaccine responses has been done with vaccines of other pathogens. Nonetheless, these studies are instructive of the potential for commensal microbe-driven individual variations that might inform future OCV strategies and design. Rotavirus is a prevalent gastrointestinal viral infection that results in significant childhood mortality and is a major component for hospitalized gastroenteritis cases.^238^ There are currently two main attenuated rotavirus vaccines: RV5 (Rotateq) and RV1 (Rotarix). Rotateq is a pentavalent bovine strain, and Rotarix is a live attenuated monovalent human rotavirus vaccine. Rotateq was very effective in a European population, as it was 98.3% effective against severe rotavirus gastroenteritis.^239,240^ However, when the clinical efficacy was tested in patients in Bangladesh and Vietnam, the overall efficacy was 64% in Vietnam and 43% in Bangladesh.^241^ A recent study compared age-matched rotavirus vaccine responders and non-responders in rural Ghana with Dutch infants and showed that vaccine responses were highly associated with gut microbial compositions.^242^ In particular, there was an increased Streptococcus and reduced Bacteroides species in Ghanaian children with poor responses to rotavirus vaccination compared to the Ghanaian responders and healthy Dutch infants, though whether these microbiome differences were causal of divergent vaccine responses remains to be determined. A subsequent study sought to further define rotavirus vaccine immunogenicity by targeted antibiotic usage in adults.^220^ While anti-RV IgA titer did not vary over time, treatment with vancomycin led to an increase in Proteobacteria and an increase in anti-RV IgA at day 7.

Several studies have directly implicated microbiota structure in vaccine responses. Oh et al. showed that the murine microbiota is able to drive responses to vaccines in a flagellin-dependent manner; flagellin-sensing by TLR5 is necessary to stimulate immune responses in the trivalent influenza vaccine (TIV).^216^ TLR5 is not known to be a viral sensor, rather it is known to sense bacterial flagella. Initially, they tested whether TLR5 plays a role in viral vaccine immunity via *Tlr*5 ^−/-^ mice. As compared to the wild-type, there were significant reductions in TIV-specific antibodies in the TLR5 deficient mice. Since TIV did not directly stimulate *Tlr5*, antibiotic-treated and germ-free mice were immunized to understand the effect the microbiota may have on the antibody response. Accordingly, compared to wild-type mice, TIV-specific IgG decreased significantly to levels comparable to the *Tlr*5 ^−/-^ mice. A similar effect was observed in another study that showed that use of flagellin as a TLR5 agonist during vaccination resulted in increased IgG responses as well as a significant increase in influenza virus-specific T cells.^217^ A subsequent study compared antibody responses in human volunteers who were given antibiotics prior to TIV vaccination. Interestingly, vaccine-specific IgG1 responses were dampened in participants who were given antibiotics and had low preexisting titers.^218^ While the role of the microbiota has been shown to modulate viral vaccine responses, the effects of microbiome manipulation on responses to bacterial vaccines such as OCVs are still to be determined and represent an important avenue of future research.

#### Probiotics and vaccine responses

Modulation of the immune response against a live oral rotavirus vaccine has been reported with the probiotic *Lactobacillus casei* strain GG.^243^ Probiotic administration improved humoral immune responses; infants that received LGG or placebo showed a higher rate of rotavirus seroconversion. The whole cell killed cholera vaccine Dukoral has also been used in conjunction with various probiotic strains such as *Bifidobacterium lactis* and *Lactobacillus acidophilus*.^244^ Blood and saliva samples from human patients were analyzed and there was a significant increase in serum IgG at day 21 post-infection in patients receiving the previously mentioned probiotic strains as compared to the placebo. However, there were no differences in serum IgA or IgM. As shown by several studies, microbial colonization of the gut is an important immunomodulatory factor for stimulating IgA responses in the gastrointestinal lumen.^245,246^ For example, bacterial metabolism of bile acids has shown a role in promoting the differentiation of T_reg_ and T_H_17 cells, which are crucial for maintaining intestinal homeostasis.^247–249^ The effects of these interactions still remain to be elucidated in relation to protection against *V. cholerae* (Figure 5). Diet is also a strong modulator of gut microbial communities, and as malnutrition is often a co-morbidity in cholera endemic regions, nutrition as a driver of microbiome structure leading to immune responses to cholera infection or vaccination is an important avenue for research. Studies by the Gordon lab identified promising nutritional supplements that consist of various local food sources such as spirulina, amaranth, flaxseed, and micronutrients, with the aim to curb childhood malnutrition.^177^ Germ-free mice were given fecal microbial transplants from malnourished Bangladeshi children along with nutritional supplementation and immunized orally with cholera toxin. Gut microbiomes responsive to nutritional supplement exhibited increased CT-specific fecal IgA antibody, and were capable of invading hyporesponsive microbiomes and augment CT-specific immune responses. While there are various studies that show the promise of probiotics as vaccine adjuvants, there are also conflicting studies that suggest limited impact on vaccine efficacy. A study by Matsuda et al. investigated whether *Bifidobacterium breve* strain Yalkult (BBG-01) enhances immunogenicity of an oral cholera vaccine for children in Bangladesh. In the healthy children aged 2–5 y, *Enterobacteriaceae* count was significantly lower in the BBG-01 group than in the placebo, but vibriocidal antibody responses were similar.^250^ These divergent results highlight the need to further examine the role of specific microbiota members in modulating host immune responses to *V. cholerae* and OCVs.

## Perspectives and future directions

Despite advances in treatment, cholera remains a serious global health challenge. Even with control of mortality as a result of effective oral rehydration therapies, the high morbidity associated with cholera demands new therapeutic and prophylactic approaches. The increasing rate of antibiotic resistance in *V. cholerae* also suggests that approaches targeting virulence gene regulation and nutrition using the gut microbiome, which are less likely to engender pathogen resistance mechanisms, may be required for cholera control. Over the last decade, many large-scale metagenomic studies have demonstrated that great bacterial genetic diversity exists between people of different cultures and geography, underpinning the gut microbiome’s potential to variably influence numerous phenotypes from nutrition to infection to immune responses.^5,10,11,213^ A growing body of research, only a subset of which can be reviewed here, has established several mechanisms and lays the foundation for future studies linking microbiome structure/function and *V. cholerae* interactions with the host and host-associated commensal microorganisms (Figures 1, Figure 2). Given the temporal, geographical, and inter-individual diversity of microbiome and microbial functions in the gut, the biology of commensal microbes may serve as a personalized susceptibility factor for *V. cholerae* infection, pathogen fitness *in vivo*, and ultimately host responses to infection and vaccination.

The study of interactions between commensal microbes and gastrointestinal pathogens such as *V. cholerae* is a growing field. While *V. cholerae* displays great genomic diversity,^251,252^ the majority of studies of commensal–pathogen interaction have focused on O1 serotype biotype El Tor strains. Recent variants of *V. cholerae* that now account for many of the currently observed cholera cases display a combination of traits from the two main pathogenic biotypes, Classical and El Tor, including more severe diarrhea and increased levels of T6SS expression.^48^ These factors may exhibit different pathogen/commensal interaction behaviors compared to older El Tor lineages in experimental models.

Key to studies on interactions between the microbiome, *V. cholerae*, and the host are tractable experimental model systems to identify candidate microbes with anti-pathogen activity and to test targeted microbial modification approaches. Germ-free mice, or animals treated with antibiotics to deplete native murine microbes,^7,26,46,48,253^ can serve as hosts for either complete human fecal microbiomes or defined consortia of culture isolates. These systems allow for the addition, removal, or specific formulation of mixes of microbes to determine their effects on both pathogen fitness and virulence gene expression. These experimental approaches also allow for studies addressing a key consideration in developing new probiotics: the consistency of active effects across many microbial backgrounds. Combinatorial approaches using randomized microbial consortia in systems with controlled microbial content have been used to determine whether specific microbes exert effects *in vivo* even when the background of other colonizing microbes varies, including with *V. cholerae* colonization resistance.^46,254^

Several broad classes of strategies have been developed to target the microbiome for a variety of important phenotypes: probiotic approaches focus on identifying beneficial microbes, while prebiotic approaches focus on the use of dietary or other supplementary compounds to boost the growth of beneficial microbes. Several studies have focused on the role of preexisting probiotic organisms in affecting *V. cholerae*. A commonly studied probiotic *Lactobacillus* species was shown to have antibacterial activity against *Vibrio* species.^255^ When pretreated with *Lactobacillus acidophilus*, Caco-2 epithelial cells have increased cell viability; the adherence, internalization, and cholera toxin expression of *V. cholerae* are also repressed.^256,257^ Other probiotic approaches have focused on controlling *V. cholerae* by acidification of the gut environment, for example, in the use of engineered *Lactococcus lactis* strains that are able to both act as a sensor of *V. cholerae* infection via detection of *V. cholerae*-specific autoinducers and limit pathogen colonization via decreases in intestinal pH.^258^ However, some studies have found that existing probiotic species, often selected for their fitness in specific fermented dietary preparations, may be very limited in their ability to transfer into human microbiomes and thus mediate their anti-*V. cholerae* prophylactic effects in the absence of repeated inoculation.^253^ As our understanding of microbial interactions and biochemical functions in the microbiome increases, several studies have established in principle that bacteria can be engineered to target specific *V. cholerae* molecular pathways *in vivo*, including the production of virulence-suppressing QS signals,^7,121^ and metabolism of virulence-activating signals such as host-derived bile.^46^ That these functions can be mediated by native gut commensals that have co-evolved with human populations suggest that by identifying human gut microbiota members with bioactive properties against pathogens, we are also isolating candidate next-generation probiotics that are able to stably colonize the gut and mediate beneficial functions over time.

Microbiome structure is strongly driven by the diet of the host.^6,150,259,260^ However, the role of specific nutrient sources and the interaction of commensals and *V. cholerae* during infection is not well understood, including the key nutrient sources used by *V. cholerae* for rapid expansion *in vivo* and how commensal microbes shape the gut nutrient landscape for *V. cholerae*. A better understanding of these factors may guide the development of targeted nutritional prebiotic interventions to drive specific effects on gut microbiome and concomitant *V. cholerae* colonization resistance.

Ultimately, a better understanding of the microbiome may also yield durable approaches for cholera control targeting the host. Mucosal vaccines continue to be an integral prevention strategy for several GI pathogens, including rotavirus and *V. cholerae*. Yet, vaccination outcomes diverge based on geographical locations, potentially due to variability in gut microbiome composition.^221–223^ Tantalizing research has identified microbial correlates to vaccine immunogenicity and efficacy for other gastrointestinal pathogens, but the relationship of specific microbiome configurations and immune responses to *V. cholerae* infection and OCV administration has not been elucidated mechanistically. By better understanding the contributions of commensal microorganisms to these phenotypes, novel microbe-targeted strategies may be developed to promote even and robust host immunity against *V. cholerae*.

The gut microbiome sits at the nexus of a complex network of interactions between pathogens, gut chemical microenvironment, microbial and host nutrition, and host immunity. By attaining a better mechanistic understanding of these networks, we may be able to develop rationally designed probiotic and prebiotic strategies for the durable control of *V. cholerae* and cholera in human populations.
